# Supercritical fluid technology for solubilization of poorly water soluble drugs via micro- and naonosized particle generation

**DOI:** 10.5599/admet.811

**Published:** 2020-06-29

**Authors:** Shashi Kiran Misra, Kamla Pathak

**Affiliations:** 1 University Institute of Pharmacy, CSJM University, Kanpur, 208026, India, Email: shashisarthak@gmail.com; 2 Faculty of Pharmacy, Uttar Pradesh University of Medical Sciences, Saifai, Etawah 206130, India

**Keywords:** Supercritical fluid techniques, Micronization, nanonization, solubility, bioavailability, composite particles

## Abstract

Approximately two-third of the compounds in the pharmaceutical industry were developed through combinatorial chemistry and high throughput screening of particulate solids. Poor solubility and bioavailability of these pharmaceuticals are challenging attributes confronted by a formulator during product development. Hence, substantial efforts have been directed into the research on particle generation techniques. Although the conventional methods, such as crushing or milling and crystallization or precipitation, are still used; supercritical fluid technology introduced in the mid-1980s presents a new method of particle generation. Supercritical fluid processes not only produce micro- and nanoparticles with a narrow size distribution, they are also employed for the microencapsulation, cocrystallization, and surface coating with polymer. Recognized as a green technology, it has emerged as successful variants chiefly as Rapid Expansion of supercritical solutions (RESS), Supercritical anti-solvent (SAS) and Particles from Gas Saturated Solution (PGSS) depending upon type of solvent, solute, antisolvent and nebulization techniques. Being economical and eco-friendly, supercritical fluid technolgy has garnered considerable interest both in academia and industry for modification of physicochemical properties such as particle size, shape, density and ultimately solubility. The current manuscript is a comprehensive update on different supercritical fluid processes used for particle generation with the purpose of solubility enhancement of drugs and hence bioavailability.

## Introduction

In the last decade, supercritical fluid (SCF) processes have been extensively utilized for pursuing chemical reactions, extraction, crystallization, precipitation, purification, and development of micro- and nanoparticles. Considerable research efforts are being made to modify solubility and improvement of bioavailability of poorly soluble drugs through SCF. As a well known fact the bioavailability of drugs depends on the absorption through gastrointestinal tract that is in turn governed by their solubility and dissolution. Thus the particle size is of utmost importance. Conventional methods such as crushing, milling, crystallization and precipitation are commonly utilized for particle development in the pharmaceutical industry. Each of these methods has their own set of limitations. SCF technology presents an innovative approach for particle formation that evades most of the downsides related to the traditional methods [1]. Hence, the technology has firmly positioned itself in the pharmaceutical arena. SCF finds a vital application in the development of dry powder inhalers (mean particle size of 2 to 5 μm) that accurately deliver precise dose to the lungs. Furthermore, SCF technology can be explored for the development of sustained and controlled release systems via microencapsulation [2, 3], coating and formation of composite particles [4]. This technology is eco-friendly, green process that generates less waste during operation and produces fine product at minimum cost.

**Figure 1.**
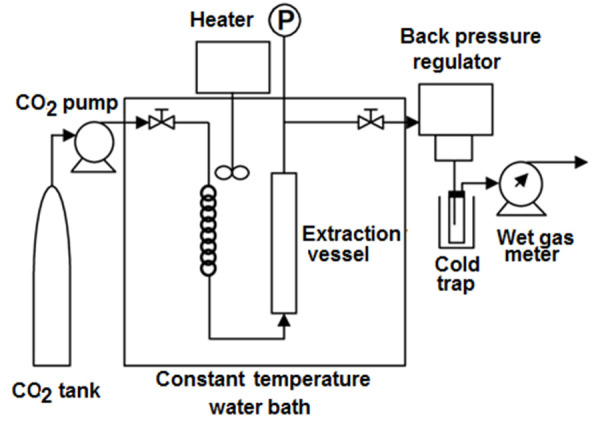
Scheme of the SCF technology process

The accuracy presented by SCF processes permits micronization of drug particles, often to submicron stage. The SCF processes can generate nanosuspensions of particles to the tune of 5-2,000 nm [5]. The technology acts as a re‐precipitation aid for rapid and uniform nucleation of the solute in all its variants of fine particle formation. The performance efficiency of this technology is based on proper solvent selection and by adjusting critical parameters (temperature and pressure) during operations (Fig. 1).

As documented in the literature, Rapid Expansion of Supercritical Solutions (RESS), Supercritical Anti Solvent (SAS) and Particles from Gas Saturated Solutions (PGSS) are the frequently employed methods not only for the fabrication of monodisperse fine powders, but also to control crystal polymorphism. It is also established that RESS can be used for CO_2_ soluble molecules, while SAS can process non-soluble molecules. However, the selection is not so simple and a good knowledge of operating conditions and phase equilibrium thermodynamics is required.

SCF methods not only produce micro- and nanoparticles of uniform size distribution, but are extensively applied for microencapsulation and polymeric surface coating on drug crystals, cocrystallization with excipients and development of soluble complexes with cyclodextrin. Nijlen *et al*. [6] demonstrated significant reduction in particle size of artemisinin and improved dissolution rate when processed with SCF technology. Table 1 lists various SCF techniques that can be used for solubility enhancement based on particle generation.

SCF processes find vital applications in almost all drug delivery routes, such as oral, intravenous, ophthalmic, pulmonary, transdermal, and implants. Revercheon *et al*. [7] emphasized the construction of various nanostructures i.e. nanofibers, nanotubes, nanowires, nanoparticles and other nano-constructions using supercritical fluid-based techniques. Byrappa *et al*. [8] explored the adaptive properties of SCF for the synthesis of advanced nanomaterials including carbon nanotubes, fullerenes, magnetic particles, quantum dots, phosphors, nanocomposites (peptide/hydroxyapatite), and gold nanoshells for drug delivery and other biomedical applications such as imaging, sensing, and cancer theranostics. The review elaborates the applications of Rapid Expansion of Supercritical Solutions (RESS), Supercritical Anti Solvent (SAS) and Particles from Gas Saturated Solutions (PGSS) for the generation of micro- and nano sized drug particles and composite particles. The write up also entails other SCF technologies that have been used for solubility enhancement of poorly water soluble drugs.

## RESS for particle generation

**Figure 2.**
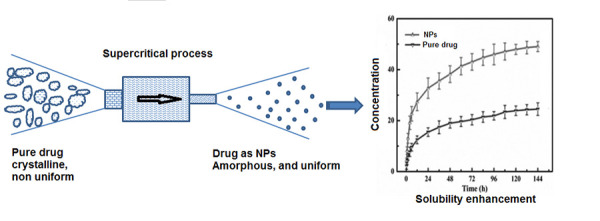
RESS process depicted diagrammatically for solubility enhancement of a hypothetical drug

RESS is composed of two steps, first to dissolve the solid compound in a supercritical fluid, and the second step results in the formation of particles by the virtue of supersaturation. The supercritical fluid (CO_2_) is allowed to pump at required pressure and temperature to the solid substance contained in the extraction chamber. The supercritical fluid expands adiabatically in the vessel, triggering a downfall of temperature and pressure, leading to the formation of fine particles [9, 10]. Hasty expansion of the supercritical solution causes reduction in the density and hence particle precipitation with minimum residual solvent occurs (Fig. 2).

Charoenchaitrakool *et al*. [11] aimed at micronization of racemic ibuprofen and examined the dissolution rate of the micronized product in a buffered solution. The solubility data at various temperatures was modeled using Peng-Robinson equations of state with Vander Waals mixing rules. The solubility of (S)-ibuprofen exhibited solubility in CO_2_ similar to (R)-form. The aggregated particles were easily dispersed by ultrasonication in water. The degree of crystallinity was slightly decreased and the intrinsic dissolution rate was higher than the original form. Likewise the amorphous nanoparticles of cefuroxime axetil were produced directly by RESS technology, without any additive. The nanoparticles obtained were between 158 and 513 nm. More than 90% of the nanoparticles dissolved in 3 min and complete dissolution occurred within 20 min, while the commercial drug achieved only about 50% dissolution in 60 min [12].

Bioavailability of the pharmaceutical substances is very important for their activity. In case of necessity, bioavailability can be improved by reducing the particle size of the drugs. Hezave and Esmaeilzadeh [13] aimed to manufacture fine particles of diclofenac and optimize the RESS conditions for generating uniform particles. Micronization resulted in the average particle size between 10.92 and 1.33 μm. The morphology of the processed particles changed to quasi-spherical while the virgin particles of diclofenac were irregular. Similarly, the reduction in particle size of digitoxin was achieved by Atila *et al*., [14] and response surface method was used to optimize the process parameters. The particle size of digitoxin was decreased from 0.2-8 μm to 68-458 nm by RESS technique and 97% of the particles were below 200 nm depending on the different experimental conditions.

The solubility of the drug substance in supercritical CO_2_ has a major effect on the average diameter of the particles prepared by RESS process. This was proved by Kim *et al*. when they used RESS for the preparation of ultra-fine drug particles using supercritical CO_2_, with no organic solvent. Three drug substances (lidocaine, griseofulvin, benzoic acid) with different solubility in supercritical CO_2_ were used, and orifice disks and capillary tubes were adapted as an expansion device. The solubilities of drug substances in supercritical CO_2_ and the effects of various operating parameters on the characteristics of the particles prepared by RESS process were experimentally investigated. The solubility of the drug substance in supercritical CO_2_ had a major effect on the average diameter of the particle prepared by RESS process, and the particle diameter decreased with the solubility for all the drugs and operating conditions [15]. In another report, the particle size of raloxifene was reduced from 45 μm to 19 nm by RESS process, the smallest of which was 18.93 nm. In addition, dissolution rate study indicated that a 7-fold increase in dissolution rate could be obtained by particle size reduction of raloxifene using RESS. Response Surface Methodology was used for the optimization of the results and it was demonstrated that the smallest particle size could be achieved at a temperature of 50 °C, pressure of 17.7 MPa and a spray distance of 10 cm [16].

In addition to particle size reduction, RESS can also be used to achieve microencapsulation and surface coating of an active substance particle with a polymer or co-crystallization with excipients or host molecules like cyclodextrins. Kim *et al*. [17] used RESS process to produce polymeric microparticles loaded with naproxen for drug delivery applications. Poly (L‐lactic acid) (L‐PLA), naproxen, and a mixture of naproxen and L‐PLA were dissolved in supercritical CO_2_ and precipitated by the RESS process. Composite particles appeared as a naproxen core encapsulated in a polymer coating. Mishima *et al*. [18] reported a new method — Rapid Expansion from Supercritical Solution with a non-solvent (RESS-N) for forming polymer microparticles containing proteins such as lysozyme and lipase. A suspension of protein in CO_2_ was made that contained a cosolvent and a dissolved polymer amongst poly (ethylene glycols), poly (methyl methacrylate), poly (L-lactic acid), poly (DL-lactide-co-glycolide) and PEG–poly(propylene glycol) (PPG)–PEG triblock copolymer. The solubilities of these polymers in CO_2_ increased significantly with low-molecular-weight alcohols as cosolvents. The wide applications of RESS namely micronization and nanonization of APIs and generation of composite particles is further summarized in Table 2.

## SAS process for particle generation

The SAS process is a highly useful for the micronization and nanonization of synthetic drugs and natural compounds. This method refers to the precipitation of compounds in a provided supercritical fluid. The selected supercritical fluid should be essentially miscible with solvent whereas the solute should be insoluble in supercritical anti-solvents. To process SAS, selection of solvent depends upon two types of requisites, first is good miscibility with CO_2_ i.e. ethanol, acetone, toluene; and second is the solubility of solute to be precipitated. Indeed, the solvent must usually belong to class 3 (non-toxic) of the pharmaceutical guidelines. In any case, the amount of residual solvent in the crystallized powder must not exceed 5000 ppm.

Many pharmaceuticals have been processed using SAS and derived processes (SEDS, PCA, ASES). A very broad range of molecules can be used namely antibiotics, proteins, biopolymers, paracetamol, salbutamol, naproxen, ascorbic acid etc [28]. Kordikowski *et al*. [29] worked with sulfathiazole, a compound that exhibits five different polymorphs. Using a semi-continuous SAS process with methanol and CO_2_, the researchers were able to produce pure polymorph by controlling the flow rate of methanol and the temperature. Three pure polymorphs I, III and IV could be obtained by choosing the right temperature while the flow rate, and the ratio of methanol: CO_2_ had less influence on the polymorphs. The method is widely studied today because of its potential industrial applications.

A semi-continuous SAS precipitation has been used to produce rifampicin micro- and nanoparticles with controlled particle size and particle size distribution; using different liquid solvents. The best micronization results were obtained using dimethyl sulfoxide at 400 °C. The nanoparticles with mean diameter ranging from 0.4 to 1 μm were obtained at a pressure of ≥ 120 bars, while microparticles with mean diameter ranging from 2.5 to 5 μm were obtained at pressures between 90 and 110 bars. The morphology of rifampicin precipitates was different too. Nanoparticles connected in small aggregates were obtained at pressures higher than 120 bars, whereas, spherical single microparticles were obtained at lower operating pressures [30].

In a research work, a swirl mixer was employed to produce the micronized curcumin with polyvinyl pyrrolidone (PVP) by the SAS process to improve the bioavailability of curcumin. The effects of operating parameters such as curcumin: PVP ratio, feed concentration, temperature, pressure, and CO_2_ flow rate were investigated. The result showed that the optimal condition for the production of curcumin-PVP particles were at curcumin:PVP ratio of 1:30, feed concentration of 5 mg/mL, temperature of 40 °C, pressure of 15 MPa, and CO_2_ flow rate of 15 mL/min. Curcumin-PVP particles (< 150 nm) were completely soluble in aqueous solution to form a clear yellow solution unlike poorly soluble raw curcumin. The solubility of curcumin-PVP particles was 2.34 μg/mL whereas that of raw curcumin was 0.006 μg/mL after 12 h. The reason attributable was that the water-soluble polymer (PVP) can modify the surface properties of the particle and thus enhance the solubility of curcumin in aqueous solution [31].

SAS process was used for telmisartan (BCS class II drug) in a variety of ways including micronization, amorphization and solid dispersion. Solid dispersions were prepared using HPMC and PVP at 1:0.5, 1:1, and 1:2 weight ratios of drug to polymer, and pure telmisartan was also treated using the SAS process. After the SAS process, all samples were converted to the amorphous form and were hundreds nm in size. Solubility and dissolution rate were increased compared to the raw material. Though the drug’s solubility increased with increase in the amount of polymer used; the dissolution rate decreased with increasing polymer concentration. Processed pure telmisartan showed higher drug release than its original form, even though it had lower solubility compared to other solid dispersions. The authors concluded that that after controlling the formulation of solid dispersion, the SAS process could be a promising approach for improving the solubility and dissolution rate of telmisartan [32].

The SAS process was used to modify the solid state characteristics of fluconazole by preparing its polymorphs by varying the temperature, pressure and solvents. Fluconazole anhydrate form I was obtained at low temperature (40 °C) and anhydrate form II was obtained at high temperature (80 °C). Not much difference was found in solubility of the polymorphs [33]. The same research group improved dissolution rate of poorly water-soluble drug, cilostazol, using SAS process. In particular, the mean particle size and distribution were markedly influenced by drug solution concentration during SAS process. Moreover, the drug did not change its crystal form and the operating parameters probably controlled the 'crystal texture'. The micronized particles exhibited a 6.5 fold increase in dissolution rate compared to the unprocessed cilostazol [34]. Likewise, amorphous SAS treated nanoparticles of atorvastatin calcium showed increased bioavailability of atorvastatin owing to their nano-dimensional size that offered high solubility and increased dissolution rate. The oral absorption of amorphous atorvastatin calcium nanoparticles in rats was obviously higher when compared with the crystalline atorvastatin calcium after a single dose of 25 mg/kg. The AUC_0-12h_ of the amorphous atorvastatin calcium nanoparticles (179 nm) was 2.1 times that of unprocessed drug. Thus, the SAS process for generation of amorphous atorvastatin calcium nanoparticles is a promising method for enhancing their bioavailability [35].

The SAS process can also be utilized to establish co-precipitation of two different compounds and to form beta cyclodextrin complexes of poorly soluble drugs. The inclusion complex of apigenin-hydroxypropyl -beta-cyclodextrin was prepared through a SAS process and its bioavailability was evaluated. The inclusion complex exhibited improved wettability and the dissolution of apigenin inclusion complex was significantly enhanced. The inclusion complex demonstrated an enhanced oral bioavailability of approximately 6 fold when compared to pure apigenin, in rats [36]. Table 3 summarizes few additional applications of SAS process in improving solubility, dissolution or drug release of certain poorly soluble pharmaceuticals.

**Figure 3.**
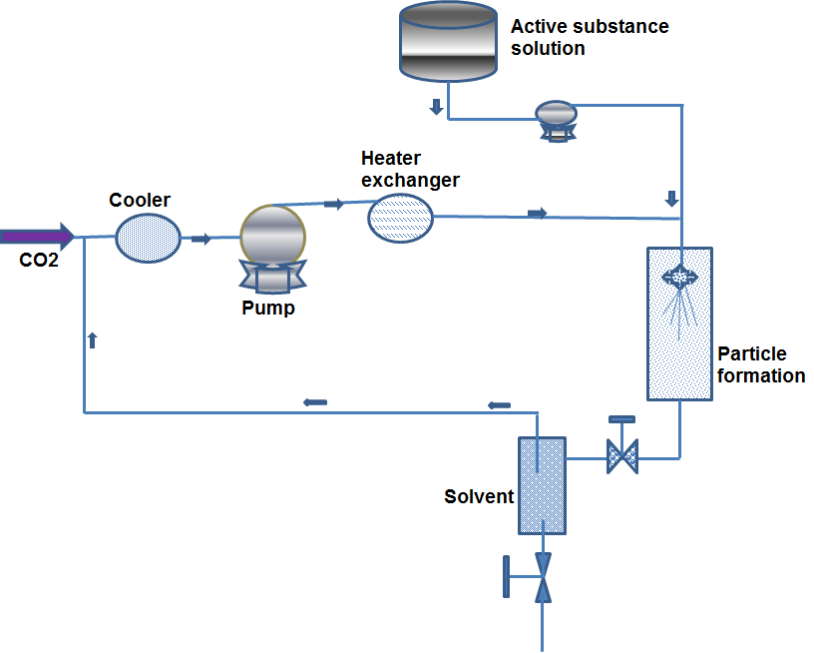
Operational design of SEDS for the formation of micronized particles

The SAS technology offers reasonable morphology, high drug loading and improved bioavailability but occasionally leads to particle aggregation that can be resolved by use of ultrasonic processor [43]. Among the various SAS based micronization techniques reported, the SEDS (solution enhanced dispersion by supercritical fluids) is an efficient process for the development of uniform sized nanoparticles [44]. York and Hanna developed modified SAS or SEDS process in 1996. This revised technique curtails the drawbacks and limitations of SAS and produces completely dried, uniform, narrow sized particles with low residual organic solvent [45]. In this modified SAS system, initially the drug and excipients are allowed to dissolve in an appropriate organic solvent followed by rigorous agitation. Thereafter, the blended components come in contact with the supercritical fluid. Especially designed coaxial nozzle in SEDS process efficiently sprays the components at high pressure that results in micronized uniform particles (Fig. 3).

SEDS has been employed to enhance dissolution of baicalein via micronization of baicalein. SEDS reduced the particle size from 25.335 μm to 0.614 μm and the particle morphology was transformed to flakes in comparison to original rod shaped crystals. The *in vitro* release studies demonstrated 50% of the drug release in approximately 20 min, and over 80% of the baicalein was released in 60 min. However, the original powder exhibited a much slower dissolution rate, and less than 25% of the baicalein was released in 60 min. The faster dissolution rate of baicalein microparticles was attributed to the reduced particle size of the baicalein and the extremely large specific surface area [46]. Likewise, the micronization of resveratrol via SEDS enhanced its solubility by approximately 2.8 times and the dissolution rate by 1.8 fold. The antioxidant efficacy of the resultant product was also enhanced significantly [47]. SEDS has been investigated to enhance solubility of bixin [48] and the dissolution rate of aescin by 5.5. fold [49].

The process can also be utilized for development of solid dispersion of poorly water soluble drugs. Water-soluble PVP and astaxanthin nanocoprecipitates were successfully prepared by SEDS precipitation. It was found that the operating pressure, temperature, PVP:astaxanthin ratio, and Z-isomer content of astaxanthin affected the particle size and the astaxanthin content in the coprecipitates. The researchers selectively used Z-isomers of astaxanthin as it has higher bioavailability and antioxidant capacity, than the E-isomer. The authors predicted that use of PVP-Z-isomers of astaxanthin coprecipitates would improve its functionality [50].

Lee *et al*. [51] investigated the application of SEDS to improve aqueous solubility of and rographolide through particle engineering. The sticky crude *Andrographis paniculata* extract was precipitated into powder from CO_2_-acetone system and CO_2_-acetone:ethanol (1:1 v/v) system. The modification of aqueous solubility of andrographolide was then attempted by manipulating its precipitation process. *A. paniculata* powder precipitated from CO_2_-acetone system at 150 bar and 40 °C consisted of large, irregularly shaped, less crystalline particles with the highest andrographolide aqueous solubility (two fold increment compared to crude extract). Complete dissolution of andrographolide from *A. paniculata* powder precipitated from CO_2_-acetone system was achieved within 90 min. Based on the higher aqueous solubility and dissolution of andrographolide, and different morphology observed from the less crystalline *A*. *paniculata* powder precipitated from CO_2_-acetone system, it was concluded that fewer impurities could have co-precipitated with andrographolide. Conclusively, the SEDS process offers many advantages over conventional SAS technique such as requirement of less solvent, applicability for thermosensitive materials and less concentration of residual solvent in the product.

## PGSS for particle generation

**Figure 4.**
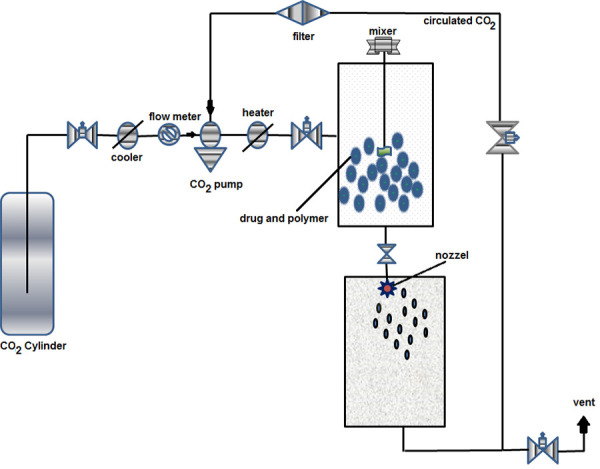
Schematic illustration of working of PGSS (Particles from Gas Saturated Solution)

PGSS is a favorable technique for polymeric encapsulation of drugs, proteins and peptides as fine particles without employing organic solvents. In this process, carriers such as polymers are melted with the dissolved or suspended active pharmaceutical ingredient (API) contained within them (Fig. 4). The resulting product is then equilibrated with supercritical-CO_2_ and expanded through a nozzle in an expansion chamber in order to form fine and porous composite particles [52]. Several carriers and APIs have been micronized using a PGSS process [53, 54]. For example, ibuprofen has been successfully micronized with different carrier materials such as polyethylene glycol (PEG) 6000 [55], poloxamers, Gelucire1 and glyceryl monostearate [56]. PEG 4000 has also been used as a carrier for the micronization of poorly water-soluble drugs [57].

Another particle generation process close to PGSS has been described by Sievers *et al*. [58] that consists of production of a dense fine droplet aerosol plume followed by a drying step. The aim of this process was to obtain fine particles usable in a dry powder inhaler form. This patented process has been used with lactose, for developing dry powder inhaler of anti asthamatic drugs: albuterol sulfate and cromolyn sodium. The product comprised of fine spherical particles in the range of 0.1– 3 μm making them suitable for inhalation.

In the experiments carried out in a pre-expansion pressure range from 100-200 bar, the particle size of nifedipine was reduced from 50 to 15 μm. At higher pressures smaller particles were formed. With the particle size reduction the dissolution rate increased to some extent, but the anticipated effective surface area was probably reduced by the drug's hydrophobicity and agglomeration of the particles during micronization [59]. Pestieau *et al*. [60] optimized PGSS process for a fenofibrate lipid-based solid dispersion formulation. The researchers aimed to develop a formulation containing fenofibrate and Gelucire1 50/13 (Gattefossé, France) in order to improve the oral bioavailability of the drug. The PGSS process was optimized according to the *in vitro* drug dissolution profile obtained using a biphasic dissolution test. Based on the fact that the propensity of drug precipitation during in vitro testing of lipid-based formulations can serve as a potential indicator for the *in vivo* performance of a drug, the authors predicted less time to reach *C*_max_ and its sustainment for longer period for PGSS derived solid dispersion than conventional solid dispersions. The authors also deduced that these formulations may avoid the precipitation of this poorly water- soluble drug *in vivo*. Furthermore, an increase in apparent solubility induced by the formulation used can lead to an enhancement of a drug's permeability through biological membranes. Thus, improvement of the oral bioavailability of fenofibrate should be more pronounced with the PGSS formulation as a result of supersaturation being maintained for longer period.

Fenofibrate solid dispersions were also investigated by Krananja *et al*. [61]. The authors used PGSS process for the carrier materials: Brij 5100 and polyethylene glycol PEG 4000, for the incorporation of the insoluble drugs nimodipine, fenofibrate, and o-vanillin with the purpose of improving their bioavailability and dissolution rate. The authors however, reported primarily the influence of processing parameters of PGSS on process yield, particle size distribution, loading efficiency and dissolution rates. The general conclusion was that with increasing pre-expansion pressure, the mean particle size of nimodipine-Brij S100, vanillin-Brij S100, and vanillin-PEG 4000 solid dispersion(s) decreased. In the case of a mixture of fenofibrate-Brij S100, the anticipated effective surface area was slightly reduced with pressure as a result of agglomeration. The loading efficiency of drugs in carriers was high and the particles obtained were irregular in shape. The authors deduced that a combination of factors, including particle size reduction and interaction between drug(s) and hydrophilic carrier(s), contributed to enhancing the dissolution rates of precipitated solid particles. On average, a 3.5-fold greater amount of nimodipine was dissolved in 1 h from solid dispersions, compared to unprocessed nimodipine. Dissolution profiles were compared with a different *f*1 factor and a similarity factor *f*2. It was confirmed that the dissolution character of processed o-vanillin and fenofibrate by PGSS was different from that of unprocessed o-vanillin and fenofibrate.

The PGSS process is not limited to production of solid dispersions; the technique also finds applications in production of a variety of composite particles namely solid lipid particles, microparticles and microcapsules that have potential to modulate drug release and thereby bioavailability. Table 5 presents a cross section of such research reports.

PGSS process is simpler in operation than other techniques as the therapeutic substance need not be necessarily solubilized in the supercritical fluid (CO_2_). It requires low solvent gas supplies and pressure for operative purposes as compared to other processes. PGSS process can be employed to develop inorganic powders into multifunctional pharmaceutical compounds. However, precautions need to be taken while processing thermolabile substances.

## Other supercritical fluid techniques for enhancement of solubility

### Rapid expansion of a supercritical solution into a liquid solvent (RESOLV)

When the traditional RESS is modified by expanding the supercritical solution into a liquid solvent instead of ambient air it is termed as rapid expansion of a supercritical solution into a liquid solvent (RESOLV). This technique can be used for the production of nanoscale particles (less than 50 nm in average diameter) from a CO_2_ soluble polymer [69]. The development of versatile methods for the preparation of homogeneously distributed nanoscale drug particles and their stable aqueous suspension is still a major challenge, despite the extensive effort based on traditional techniques.

RESOLV was employed for the production of drug nanoparticles of two model drugs: ibuprofen and naproxen, which are somewhat soluble in supercritical CO_2_ and practically insoluble in water. The RESOLV process yielded aqueous suspensions of homogeneously distributed ibuprofen (average size of 40 nm in diameter and a size distribution standard deviation of 8.5 nm) and naproxen nanoparticles of average size of 64 nm in diameter and a size distribution standard deviation of 10 nm. The nanoparticles agglomerated to form larger aggregates on a longer time scale. The agglomeration can be minimized by the presence of a stabilization agent, e. g. poly (N-vinyl-2-pyrrolidone) in the aqueous suspension. The technique may serve as a “clean” way for nanosizing the drug particles and the preparation of stable suspensions thereof for formulation and other delivery related requirements, specifically addressing the bioavailability issues [70].

### Depressurization of an Expanded Liquid Organic Solution (DELOS)

Polymorphism is very common in pharmaceutical drug substances since their solubility and bioavailability are determined by the crystalline structure adopted by the solid drugs [71]. In addition, the drug substance will in most cases be handled as a solid in some stages of the manufacturing process, and its handling and stability properties may depend on the solid phase. Consequently, the control of the production of a given solid polymorph is of the utmost importance in such commercial applications and industries. In a study by Sala *et al*. [72] it was observed that the precipitation of pure monoclinic E form of stearic acid was favored by DELOS process, a kinetically controlled crystallization in which high supersaturation levels are rapidly achieved. Remarkably, the DELOS process for the first time provided a pure polymorphic monoclinic E form, without the presence of traces from other polymorphs that always are present when using other kinetically driven methods, such as the conventional fast cooling. On the contrary, the C polymorph was preferentially obtained by the thermodynamically controlled GAS technique in which the increase of the solution supersaturation is slow or low supersaturation levels are attained.

DELOS can be used as a route to obtain nutraceutical products that might show enhanced functional properties. Phytosterols are absorbed to a much smaller extent in the body compared to cholesterol and they interfere with the intestinal absorption of cholesterol. DELOS methodology has the potential to process phytosterols into micrometer or submicrometer particles, which is not possible with conventional technologies. Moreno-Calvo *et al*. [73] processed β-sitosterol through DELOS thereby reducing the crystals from 188 μm to <6.5 μm, with a narrow size distribution at all processing conditions employed. The new phase showed higher chemical purity and higher crystallinity than the native mixture. The authors recommend further studies to confirm the expected enhancement of absorption and bioavailability of β-sitosterol.

### Aerosol Solvent Extraction System (ASES)

ASES methodology has been used extensively for processing pharmaceuticals and biopolymers and is capable of producing micrometer sized or nanosized particles with low levels of residual solvent [74]. Furthermore, in the ASES process the particle size and morphology can be easily modulated by the optimization of the processing parameters. The therapeutic applications of silybin, an antihepatotoxic polyphenolic substance, are strongly limited by its poor solubility and low bioavailability. The issues can be addressed by designing nanodrug via ASES. In the process, water soluble and biocompatible PVP was used to improve the dissolution rate and bioavailability of silybin. The size of the silybin PVP nanodrug was to the tune of 100 to 300 nm. Compared with raw silybin, the nanodrug had low crystallinity and hence showed solubility enhancement by more than 8-fold and hence in dissolution too [74].

Copper-indomethacin is a non-steroidal anti-inflammatory drug currently available for veterinary use. Its application is limited to oral formulations because of its poor solubility in biocompatible solvents. Meure *et al*. [75] prepared microspheres of PVP and copper-indomethacin that ranged in size from 50 nm to 4 μm. A coprecipitate containing 10%wt copper-indomethacin and 90%wt PVP was found to be at least 93 times more soluble in ethanol than factory-grade copper-indomethacin. The significance of these results is that the coprecipitate of copper-indomethacin may be used for parenteral applications [75].

Rao *et al*. [76] suggested enhancement of the dissolution rate, apparent solubility and oral bioavailability of tadalafil by nanosized amorphous particles prepared by using antisolvent precipitation. Optimization of processing parameters yielded amorphous tadalafil solid dispersion of approximately 5-10 μm. The solid dispersion obtained using the optimized process conditions exhibited 8.5 times faster dissolution rates in the first minute of dissolution, 22 times greater apparent solubility at 10 min and a 3.67-fold increase in oral bioavailability than the unprocessed tadalafil.

### Supercritical solvent impregnation (SSI)

Among the different approaches that employ supercritical fluids for pharmaceutical purposes, polymer impregnation techniques have been recently used in literature to achieve the impregnation of many kinds of polymers namely, poly(lactic-co-glycolic acid), ethyl/methyl cellulose, poly(dimethylsiloxane), poly(methyl methacrylate, etc. with a drug [77]. In particular, reports where PVP was impregnated with some crystalline drugs (i.e. carbamazepine, ibuprofen, ketoprofen) can be found [78-80]. In these processes, the crystalline drug is dissolved by supercritical CO_2_ and thus conveyed through the swollen polymeric matrix until the partition equilibrium takes place between the phases. The encapsulated particles display extensive solubility, better diffusion and substantial dissolution profile owing to the supercritical fluid that plays a role of cosolvent.

In a study by Banchero *et al*. [81] successful impregnation was reported for all the PVP K15-piroxicam systems at 300 bar and 100 °C. Good results in terms of acceleration in the drug release were obtained with the PVP K15-piroxicam system. The best result was obtained for the impregnated sample containing a piroxicam amount equal to 11.3%, which released 94.7% of the drug after 10 min, with respect to 7.8% released by the corresponding physical mixture after the same period of time.

### Gas Antisolvent process (GAS)

In the GAS processes, a solute is dissolved in solvent and loaded into a crystallizer. The solution is then expanded by injecting carbon dioxide into the crystallizer. A sharp reduction of solute solubility in liquid phase is observed and subsequently particle precipitation occurs. This technique is used for drugs with low solubility in the supercritical fluid. The mean particle size and particle size distribution are controlled by GAS variables. Various drugs have been micronized by GAS process namely, microparticle production of carbamazepine [82], controlled crystallization of β-carotene [83], caffeine [84], phenanthrene [85] and paracetamol [86]. Control of GAS processing variables resulted in a decrease in ampicillin particle size from 359 to 260 nm. The mean particle size was 425 nm for the lowest pressure (9 MPa). When the pressure was increased, a smaller mean particle size (220 nm) was obtained. The smaller mean particle size was observed at the lower temperature and low solute concentration [87].

All the detailed SCF techniques are virtuous alternatives for micronization and nanonization of drugs that require particle engineering for modification and improvement of solubility. SCF processes are frequently utilized to formulate readily solubilized drug carrier systems i.e. microparticles, nanoparticles, inclusion complexes, solid dispersions, macromolecular powders and microporous foams. Some of these have been elaborated in Table 4.

## Conclusions

Supercritical fluid techniques for micronization and solubility enhancement have been progressively applied in pharmaceutical industry. The characteristic advantages of SCF technology include non-toxicity, eco-friendly and flexibility that make it suitable for green chemistry. SCF processes are proven promising strategies to develop and design drug delivery system of those drugs whose solubility and bioavailability is significantly low. Moreover, SCF technologies are also utilized for formulating drug carrier owing to unique solvent features that can be readily modified by altering operating temperature and pressure. Several issues still remain i.e. the influence of operating parameters on the characteristics of the particle produced (size, morphology, polymorphism), the comprehension of the fluid dynamics, the nucleation phenomenon, the crystal growth under provided conditions etc. Whatsoever, the technology has arrived and is promising green option for pharmaceutical development.

## Figures and Tables

**Table 1.** Protagonists of supercritical fluid technology

Processing Component	Process/ Acronym
Solvent	Rapid expansion of supercritical solution (RESS)
	Rapid expansion of supercritical solution into a liquid solvent (RESOLV)
	Rapid expansion of supercritical solution into an aqueous solution (RESS-AS)
	Rapid expansion of supercritical solution with a non-solvent (RESS-N)
Anti-solvent	Gas anti-solvent (GAS)
	Supercritical anti-solvent (SAS)
	Aerosol Solvent Extraction System (ASES)
	Particles by Compressed Anti-solvent (PCA)
	Solution Enhanced Dispersion by Supercritical Fluids (SEDS)
Solute	Particles from Gas-Saturated Solutions (PGSS)
	Depressurization of an Expanded Liquid Organic Solution (DELOS)

**Table 2.** A cross section of micronization and nanonization of some drugs using RESS technology

API	Objective	Research highlight	Ref
Coumarin	Coumarin nanoparticles were prepared and the effect of temperature, pressure, spray distance and nozzle diameter on particle size and solubility was assessed.	A considerable decline in particle size was observed from 40.35 μm to 21.37 nm thereby affecting solubility. Quadrupolar cubic plus association theory and perturbed-chain polar statistical associating fluid theory were applied to interpret the solubility data.	[19]
Ibuprofen, aspirin and griseofulvin	RESS was used to reduce particle size. The solubility study of poorly soluble drugs was performed employing five cubic equation of state (EoS) with two mixing rules.	Based on the calculated solubilities, two of the most accurate equations of state are PR-vdW and PR-KM with less absolute percent deviation than the other EoS for all systems	[20]
Ipriflavone	The solubility of ipriflavone was enhanced through the RESS process using supercritical CO_2._	Results outlined improved solubility of ipriflavone in supercritical CO_2_. Additionally, the particle size was reduced to 4.4 μm from the original 30.9 μm utilizing RESS process.	[21]
Letrozole	RESS with solid cosolvent (RESS-SC) was employed to precipitate nanoparticles of letrozole	Obtained findings suggested enhanced solubility of letrozole (7.1 times) in the ternary phase with solid co-solvent application in RESS process. The average particle size was reduced 30 nm to 19 nm.	[22]
Aprepitant	Effect of parameters i.e. pressure, temperature, spraying distance and nozzle diameter was studied on the nanoparticles morphology.	Significant reduction in the particle size (micrometer to nanometer) was observed for the nanoparticles developed through RESS-SC method. The dissolution rate of aprepitant was increased by 8.2 times, suggesting improved solubility of the drug.	[23]
TBTPP (5, 10, 15,20-tetrakis (3, 5-bis- tri fluoro methyl phenyl porphyrin	RESS process was investigated applying numerical modeling for particle formation of TBTPP.	Peng-Robinson EoS with Kwak-Mansoori mixing rules were applied after the optimization of pressure and temperature. The improved solubility was measured by numerical modeling and the results were compared with experimental data.	[24]
Progesterone	Fine progesterone particles were produced and the solubility was analyzed by varying temperature and pressure and compared with a well known model.	The solubility studies were correlated with empirical density-based models and the Peng-Robinson equation of state model. It was found to be improved range of 5.3 × 10^− 5^–8.9 × 10^− 4^, with submicron size.	[25]
Paracetamol	A novel fluidized-bed coating process using RESS was described for the coating of fine particles.	Microspheroidal catalyst particles (average particle size 56 μm) were used as the core particles. Supercritical CO_2_ solution of paraffin was expanded through the nozzle into the bed that was fluidized by air. The coating mass and coating rates were measured by a sampling method. A stable coating of fine particles was achieved without the formation of agglomerates at room temperature	[26]
Ibuprofen and nicotinamide	RESS was used as a means of simultaneous micronization and cocrystallization of drugs with poor aqueous solubility.	1:1 cocrystals of ibuprofen and nicotinamide with high purity were produced. The specific surface area of RESS cocrystals was increased by almost tenfold in comparison to cocrystals produced by slow solvent evaporation and the mean dissolution time of ibuprofen from RESS cocrystals was decreased. For drugs with dissolution- limited bioavailability, RESS cocrystallization may be a superior approach in comparison to established cocrystallization techniques.	[27]

**Table 3.** A compilation of reports on solubility enhancement of poorly soluble drugs affected by SAS technology

API	Objective	Outcome	Ref
Tolfenamic acid	SAS parameters were evaluated for solid state property modification and improvement of dissolution profile of tolfenemic acid.	SAS technology was efficient in modifying the solid-state. It produced microparticles with improved dissolution behavior.	[37]
*N-*acetyl-cysteine	The study aimed to micronize *N-*acetylcysteine by the anti-solvent SEDS technique.	Micronized *N-*acetylcysteine presented prominent biological activity (100 times) depicted by lower minimum inhibitory concentration compared to non-micronized *N-*acetylcysteine	[38]
Curcumin	Curcumin based dye extract was developed employing SAS. Eudragit® L100, Pluronic® 127 and tween 20 were added to improve the aqueous solubility and stability.	Formulation of a soluble curcumin was carried out for food application. Highest aqueous stability and solubility was observed at pH 4. The mean diameter and zeta potential of the amorphous curcumin particles was 5667.4 nm and 11.21 mV respectively.	[39]
Warfarin	To determine solubility of warfarin in supercritical CO_2_ using SAS	Regular crystals of warfarin with a mean particle size of 6.6 μm were produced	[40]
Irbesartan	To improve the dissolution of irbesartan through solid dispersions using SAS concept.	The crystalline state of the drug was transformed into the amorphous state. The dissolution was enhanced after formation of irbesartan solid dispersions	[41]
Azithromycin	Solid dispersions of azithromycin were developed utilizing variable amounts of PEG 6000, sorbitol, SLS and Poloxamer 188,	The amorphous solid dispersions of azithromycin demonstrated enhanced solubility with PEG 6000 and SLS.	[42]

**Table 4.** A cross section of composite particles of drugs prepared using PGSS process

API and excipients	Strategy	Result highlight (s)	Ref
Curcumin, tristearin, soyphosphatidyl-holine, DMSO	Curcumin embedded solid lipid particles were developed via PGSS with less quantity of organic solvent.	The use of helium in the process of PGSS, for designing of lipid mixture exhibited improvement in the biopharmaceutical properties and therapeutic value of curcumin.	[62]
S-(+)-ibuprofen, Poloxamers, Gelucire, Glyceryl monostearate(GMS)	PGSS was employed for the enhancement of solubility of (+)-ibuprofen using hydrophilic or hydrophobic carrier.	Spherical and porous particles (50-200 μm) with 90% encapsulation efficiency were produced. The solubility of ibuprofen was significantly enhanced with poloxamer in the gastro-intestinal fluids; gelucire and GMS did not enhance the solubility of ibuprofen.	[63]
Ketoprofen, Glyceryl monooleate (GMO), Gelucire 43/01™, Geleol™ and Gelucire 50/13™	For production of structured lipid carriers a liquid glycerolipid (GMO), was incorporated into three solid glycerolipids with hydrophilic-lipophilic balance ranging from 1 to 13 and compared with solid lipid particles.	Irregular porous microparticles with a wide particle size distribution were obtained. The drug loading capacity of the structured lipid carriers increased as the GMO content in the particles increased, achieving a maximum encapsulation efficiency of 97% for the 3:1 mass ratio. The structured lipid carriers presented an immediate release of ketoprofen from its matrix with higher permeation through a mucous-membrane model, while solid lipid particles presented controlled release of the drug with less permeation capacity.	[64]
Quercetin, soybean, lecithin, and pluronic L64	To modify bioavailability of quercetin through microencapsulation	More homogenous lyophilized less crystalline encapsulated quercetin particles were reported with enhanced bioavailability	[65]
β-carotene, poly-(ε-caprolactone)	β- carotene was encapsulated in poly- caprolactones, and precipitated out using PGSS process.	Small, regular, uniform, microencapsulated β-carotene particles in the size range of 100 –600 μm were obtained, that demonstrated enhanced solubility.	[66]
1,3-diphenyl-2-propenone (chalcone)	Microparticles of chalcone alone and with lipid carriers were developed via PGSS and the solubility was analyzed.	The lipid carriers influenced the solubility of trans-chalcone in simulated gastric and intestinal fluids, without addition of enzymes.	[67]
Omega-3 PUFA-rich salmon oil and astaxanthin	Microparticles of omega-3 PUFA-rich salmon oil in PEG-6000 were developed through PGSS concept.	Developed microparticles showed significant thermogravimetric stability up to 350 °C. Moreover, *in vitro* release of oil in fluids stimulating gastric conditions was faster than in distilled water.	[68]

**Table 5.** A cross-section of research reports on micronized particles using discrete SCF technology

SCF Process	API	Purpose	Highlight	Ref
DELOS	ibuprofen and naproxen	Micronization and determination of solubility	Ibuprofen showed same solubility profile, both in CO_2_-expanded ethanol and CO_2_–expanded acetone mixtures; whereas the naproxen solubility was greatly dependent on the nature of the solvent i.e. high in CO_2_–expanded ethanol.	[88]
RESOLV	Gambogic acid	Nanoparticles of gambogic acid were prepared to improve solubility.	Results outlined successfully preparation of nanosuspension of gambogic acid. Extended solubility data was correlated with density-based models that suggested enhanced bioavailability and antineoplastic efficacy of nanosized gambogic acid.	[89]
DELOS	Phytosterol	Nanonization and decrease crystallinity of phytosterol to modify solubility	Phytosterol nanoparticles were formulated through fast cooling, The crystallinity of the impregnated phytosterols was found to decrease in comparison to the pure phytosterol that modified water solubility.	[90]
RESOLV	Poly (l-lactide) (PLLA) nanoparticles loaded with retinyl palmitate	Nanoparticles of PLLA retinyl palmitate were developed with Pluronic F127, F68 and sodium dodecyl sulfate	Spherical PLLA- retinyl palmitate nanoparticles were prepared that possessed mean size of 40–110 nm with improved solubility and great retinyl palmitate loading.	[91]
RESOLV	Fenofibrate	Precipitation and stabilization as ultrafine particles of fenofibrate	The mean particle size was approximately 3 μm, which suggested improved solubility. The particles were found to be stable for 24 h.	[92]
GAS	Resveratrol [REMOVED HYPERLINK FIELD] and isoniazid, nicotinamide	Co-crystals of resveratrol were prepared with isoniazid and nicotinamide using CO_2_ antisolvent	The developed co-crystals exhibited enhanced bioavailability when compared to original resveratrol.	[93]
GAS	5-fluorouracil	Halloysite clay nanocarrier was developed to obtain high drug loading of 5-fluorouracil	Prepared nanocarrier loaded with 5-fluorouracil released significantly high drug release at pH 7.4 owing to improve solubility.	[94]
SSI	Quercetin	Quercetin was impregnated on Silica to enhance solubility	Several parameters i.e. temperature, time, pressure, and different cosolvents in the supercritical impregnation process were reported influential for quercetin solubility.	[95]
SSI	Promogran	Promogran was embedded on a spilanthol-enriched extract to modify solubility	Jambu extract that is completely soluble in fluid phase, was used to demonstrate enhanced solubility of promogran. A 4 h processing period was used for complete dissolution of the extract in SCF.	[96]
ASES	Irbesartan	Development of Irbesartan micro-particles and its composite micro-particles	Results highlighted modified solubility (7.5 times) and dissolution rate of Irbesatan microparticles compared to pure drug.	[97]
ASES	β-sitosterol	Preparation of submicroparticles of β-sitosterol	Powdered submicroparticles of β-sitosterol exhibited improved solubility.	[98]
ASES	Itraconazole	Preparation of solid-inclusion complexes of itraconazole with HP-β-CD	ASES-processed ITR-HP-β-CD inclusion complex solid powder showed 90% drug dissolution within 10 minutes.	[99]
